# Plaque-Charakterisierung und individualisierte Risikoeinschätzung

**DOI:** 10.1007/s00117-024-01385-y

**Published:** 2024-11-12

**Authors:** J. M. Brendel, K. Nikolaou, B. Foldyna

**Affiliations:** 1 Institut für Diagnostische und Interventionelle Radiologie, Universitätsklinikum Tübingen, Tübingen, Deutschland; 2 Cardiovascular Imaging Research Center, Department of Radiology, Massachusetts General Hospital and Harvard Medical School, Boston, MA USA

**Keywords:** Koronarstenose, Risikostratifizierung, Kalziumscoring, Hochrisiko-Plaque, Plaque-Progression, Coronary stenosis, Risk stratification, Calcium scoring, High-risk plaque, Plaque progression

## Abstract

**Klinisches/methodisches Problem:**

Risikoeinschätzung und genaue Plaque-Charakterisierung sind der Schlüssel für die individuelle Prognose der koronaren Herzkrankheit (KHK).

**Radiologische Standardverfahren:**

Standardverfahren ist die kardiale Computertomographie (CT), einschließlich des nativen Kalziumscorings und der Computertomographie-Koronarangiographie (CCTA). Die Befundung erfolgt mittels CAD-RADS-Klassifikation (Coronary Artery Disease—Reporting and Data System).

**Methodische Innovationen:**

Neue Entwicklungen umfassen die CT-basierte fraktionale Flussreserve (CT-FFR) sowie die Plaque-Quantifizierung (*virtuelle Histologie*).

**Leistungsfähigkeit:**

Ein Kalziumscore von 0 bedeutet ein Ereignisrisiko < 1 % über 10 Jahre hinweg [7, 17]. Die CAD-RADS-Klassen 1 bis 5 erlauben eine Risikobewertung im Vergleich zu Patienten ohne koronare Plaques [2]. Die CT-FFR hat eine hohe Genauigkeit („area under the curve“ [AUC] 0,90; 95 % Konfidenzintervall [KI] 0,87–0,94) in der Beurteilung der hämodynamischen Stenosenrelevanz im Vergleich zur invasiven Koronarangiographie [25]. Mittels Plaque-Quantifizierung wurde festgestellt, dass ein Anteil von über 4 % an nekrotischem Kern das 5‑Jahres-Ereignis-Risiko fast verfünffacht [29].

**Bewertung:**

Das Vorhandensein einer obstruktiven KHK (Stenose > 50 %) ist ein starker prognostischer Faktor. Die Evaluation der hämodynamischen Relevanz 40–90 %iger Stenosen mittels CT-FFR oder anderer funktioneller Tests ist in den USA bereits leitliniengerecht, jedoch noch nicht in Deutschland. Quantitative Ansätze zur Messung von Volumina und Zusammensetzung der Plaques gewinnen zunehmend an Bedeutung in der Forschung und werden voraussichtlich auch in der klinischen Praxis relevant werden.

**Empfehlung für die Praxis:**

Die Beurteilung des KHK-Ausmaßes sollte durch die CAD-RADS 2.0‑Klassifikation erfolgen, die auch Therapieempfehlungen gibt.

## Abstract (en)

**Clinical/methodical issue:**

Risk assessment and accurate plaque characterization are key to individual prognosis in coronary artery disease (CAD).

**Standard radiological methods:**

The standard of care is cardiac computed tomography (CT), including calcium scoring and coronary CT angiography (CCTA). Diagnosis is based on the CAD-RADS (Coronary Artery Disease—Reporting and Data System) classification.

**Methodological innovations:**

New developments include CT-based fractional flow reserve (CT-FFR) and plaque quantification (“virtual histology”).

**Performance:**

A calcium score of 0 indicates an event risk of less than 1% over 10 years [7, 17]. CAD-RADS classes 1 to 5 allow risk assessment compared to patients without coronary plaques [2]. CT-FFR has high accuracy (area under the curve [AUC] 0.90; 95% confidence interval 0.87–0.94) in assessing the hemodynamic significance of stenoses compared with invasive coronary angiography [25]. Plaque quantification has shown that a necrotic core greater than 4% is associated with an almost fivefold increase in 5‑year event risk [29].

**Achievements:**

The presence of obstructive CAD (stenosis > 50%) is a strong prognostic factor. The evaluation of the hemodynamic relevance of 40–90% stenoses by CT-FFR or other functional tests is already guideline-compliant in the USA, but not yet in Germany. Quantitative approaches to measure plaque volume and composition are gaining importance in research and are expected to become relevant in clinical practice.

**Practical recommendations:**

The CAD-RADS 2.0 classification, which also provides therapy recommendations, should be used to assess the extent of CAD.

Die koronare Herzkrankheit (KHK) ist weltweit eine der häufigsten Todesursachen und fordert jährlich Millionen von Menschenleben weltweit. Eine individuelle Risikoeinschätzung kann daher entscheidend sein. Die Computertomographie (CT) des Herzens bietet Informationen zur Ausprägung von Kalkablagerungen in den Herzkranzgefäßen und ermöglicht die Messung und Charakterisierung koronarer Plaques. Diese Informationen sind wichtig, um das Risiko zukünftiger Ereignisse wie Myokardinfarkte einzuschätzen.

Die nichtinvasive Bildgebung mittels Computertomographie-Koronarangiographie (CCTA) ist heute ein unverzichtbares Mittel zur Diagnose und Risikobewertung der KHK, die weltweit eine der häufigsten Todesursachen darstellt und jährlich etwa 18 Mio. Menschenleben fordert [28]. Besonders die Beurteilung von koronaren Plaques mittels CT ermöglicht die präzise Identifizierung von Hochrisikopatienten und die gezielte Anwendung präventiver Maßnahmen.

In den letzten Jahrzehnten hat sich die kardiovaskuläre Bildgebung durch fortschrittliche Technologien und Analysemethoden stark weiterentwickelt. Frühe Ansätze wie das Kalziumscoring ebneten den Weg für weitere diagnostische Verfahren wie die CCTA. Mit der Einführung von Klassifikationssystemen wie dem Coronary Artery Disease—Reporting and Data System (CAD-RADS) wurde die Auswertung der CCTA vereinfacht und standardisiert. Doch die Innovation in der kardialen CT geht weiter: Quantitative Methoden wie die CT-basierte fraktionale Flussreserve (CT-FFR) und die Plaque-Quantifizierung eröffnen neue Wege zur präzisen Charakterisierung von Plaques und zur Bewertung der Hämodynamik von Stenosen. Dies verspricht eine noch genauere individuelle Bewertung des kardiovaskulären Risikos.

## Der Pionier: Kalziumscoring

**Abb. 1:**
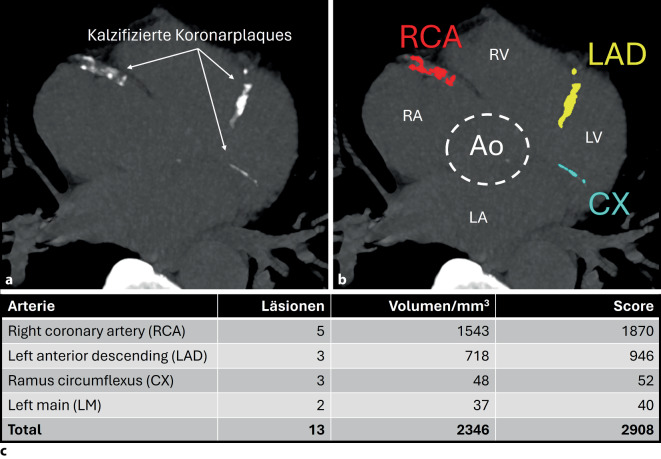
Kalziumscoring eines 74-jährigen Mannes. Multiplanare Rekonstruktionen einer nativen CT-Untersuchung veranschaulichen die kalzifizierten Koronarplaques in den Originalbildern (**a**) und die farbkodierte Kalkerkennung (**b**). *Rot *„Right coronary artery“ (*RCA*); *gelb *Ramus interventricularis anterior („Left anterior descending”, *LAD*); *türkis *Ramus circumflexus (*CX*). Der linke Hauptstamm („Left main“) ist in dieser CT-Schicht nicht miterfasst. **c** Automatische Quantifizierung und Zuordnung zu den Koronararterien; der Agatston-Score des Patienten beträgt 2908. *Ao* Aortenklappe, *LA* linkes Atrium, *LV* linker Ventrikel, *RA* rechtes Atrium, *RV* rechter Ventrikel

Das Kalziumscoring wurde in den 1990er-Jahren entwickelt. Es ist ein seit nunmehr über 30 Jahren ein bewährtes Verfahren zur Beurteilung der KHK-Ausprägung. Wie der Name der Methode verrät, wird ein Score erstellt, der die Gesamtlast kalzifizierter Koronarplaques des Patienten bestimmt. Wichtig: Nichtkalzifizierte Plaques werden nicht berücksichtigt. Praktisch erfolgt das Kalziumscoring mittels einer Niedrigdosis-CT ohne Kontrastmittel. Nach der Agatston-Methode werden Fläche und Dichte von koronaren Kalzifizierungen berücksichtigt [1], wobei als Faustregel gilt: Größere und *dichtere* Läsionen werden mit höheren Werten bepunktet (Abb. 1).

Die Summe aller bepunkteten koronaren Verkalkungen ergibt den Agatston-Score (A). Der Agatston-Score (syn. Kalziumscore) ist stark assoziiert mit kardiovaskulären Ereignissen. In großen primären Präventionskohorten wie der Multi-Ethnic Study of Atherosclerosis (MESA) und der Framingham-Heart-Studie (FHS) hat sich gezeigt, dass ein Kalziumscore von 0 mit einem niedrigen (< 1 %) jährlichen Risiko für kardiovaskuläre Ereignisse und Mortalität in den folgenden 10 bis 15 Jahren einhergeht (sog. „power of zero“; [7, 17, 27]).

Für die behandelnden Fachdisziplinen ergeben sich basierend auf dem Kalziumscore pharmakopräventive Therapieempfehlungen (Tab. 1). US-amerikanische Leitlinien verwenden den Kalziumscore bereits in der Therapieentscheidung [12].

**Tab. 1:** Kalziumscore, kardiovaskuläres Risiko und Therapieempfehlungen. (Nach [15])

Kalziumscore	Risiko	Therapieempfehlung
0	Sehr niedrig	Keine Statintherapie^a^
1–99	Gering erhöht	Moderate Statintherapie
100–299	Moderat erhöht	Moderate bis intensivierte Statintherapie + ASS 100 mg
≥ 300	Moderat bis stark erhöht	Intensivierte Statintherapie + ASS 100 mg

^a^ außer bei familiärer Hypercholesterinämie; *ASS* Acetylsalicylsäure

**Abb. 2:**
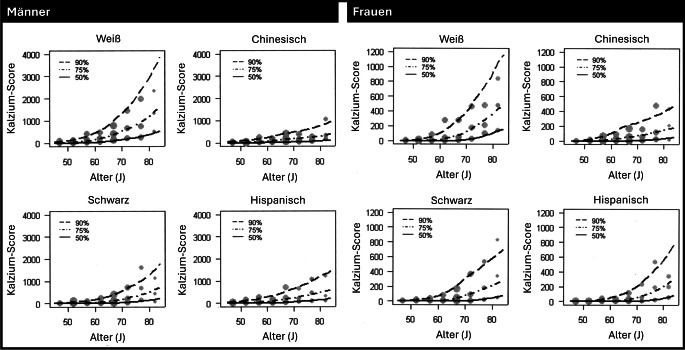
Ergebnisse der Multi-Ethnic Study of Atherosclerosis (MESA). Perzentilen des koronaren Kalziumscores nach Alter und Ethnizität für Männer (*links*) und für Frauen (*rechts*). Jedes Diagramm zeigt die geschätzten Kurven für die 50., 75. und 90. Perzentile über das Alter hinweg. Die beobachteten empirischen Perzentilen für jeden 5‑Jahres-Altersabschnitt sind als Punkte zur Referenz dargestellt. (Adaptiert nach [22])

Um den Kalziumscore in Bezug auf Alter, Geschlecht und andere demografische Merkmale zu bewerten, empfiehlt es sich, die Perzentilkurven der MESA zu verwenden ([7]; Abb. 2). Dadurch kann der individuelle Kalziumscore im Gruppenvergleich eingeordnet werden. Zum Beispiel liegt der 74-jährige weiße Mann aus Abb. 1 mit einem Kalziumscore von 2908 auf der 94. Perzentile seiner Vergleichsgruppe. Die Berechnung der MESA-Perzentilen kann online durchgeführt werden (Link in Infobox).

### Infobox Mehr Informationen zum Thema


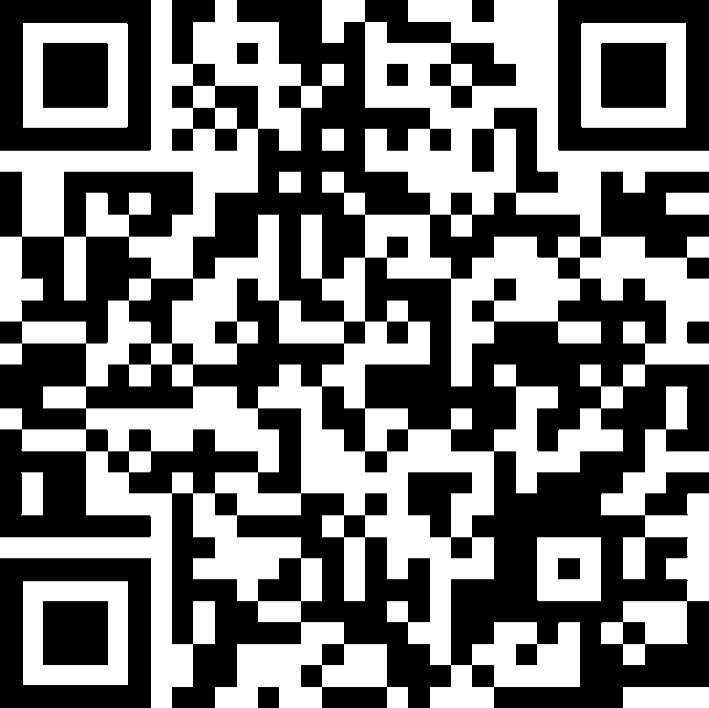


Zugriffsdatum 09.07.2024

MESA-Perzentilen: https://www.mesa-nhlbi.org/Calcium/input.aspx


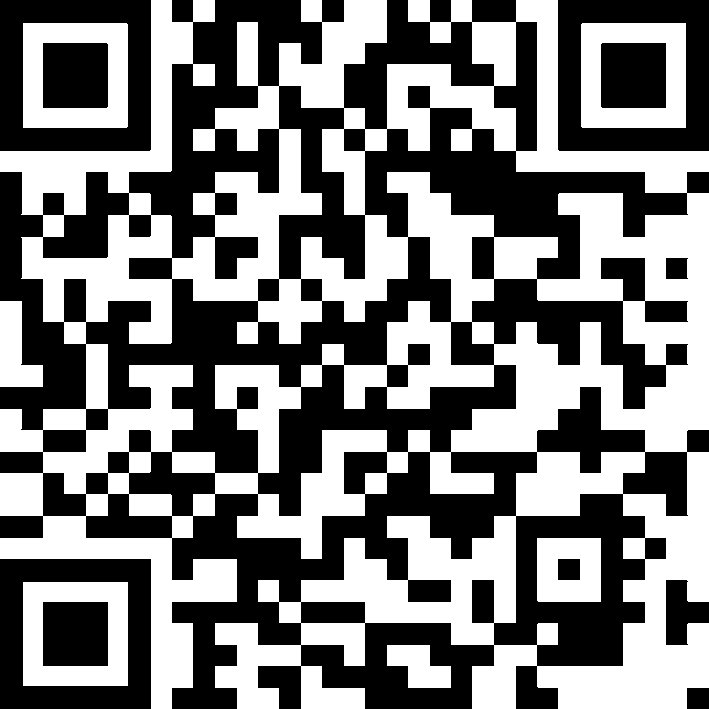


Zugriffsdatum 09.07.2024

CAD-RADS 2.0: https://pubs.rsna.org/doi/10.1148/ryct.220183

## Das Arbeitspferd: Computertomographie-Koronarangiographie (CCTA) und die CAD-RADS-Klassifikation

Die CCTA wird vor allem bei Patienten mit kardialen Symptomen durchgeführt. Durch die Verwendung von Kontrastmittel ermöglicht die CCTA die Darstellung des Gefäßlumens und eine zuverlässige Beurteilung von Stenosen. Hierbei stimmt sie gut mit der invasiven Herzkatheteruntersuchung überein. Es wurde gezeigt, dass Patienten mit stabiler Angina pectoris und mittlerer Wahrscheinlichkeit für eine Koronarstenose ähnliche kardiovaskuläre 3‑Jahres-Ereignisraten zeigen, unabhängig davon, ob sie eine CCTA oder eine Herzkatheteruntersuchung zur Abklärung erhalten (DISCHARGE Studie) [20]; untersuchungsbedingte Nebenwirkungen treten jedoch in der Herzkathetergruppe signifikant häufiger auf.

Eine große Stärke der CCTA liegt darin, dass sie Koronarstenosen zuverlässig ausschließen kann. Ihr negativer Vorhersagewert beträgt nahezu 100 %. Der Ausschluss einer Koronarstenose mittels CCTA bedeutet sogar für symptomatische Patienten ein sehr geringes Risiko (< 1 %) für kardiovaskuläre Ereignisse innerhalb der nächsten 24 Monate [18].

Ist andererseits eine Koronarstenose vorhanden, so ist dies ein wichtiger Prognosemarker, der über die klinische Risikobewertung hinausgeht. Insbesondere eine sog. *obstruktive* KHK mit einer Lumenstenose von mehr als 50 % ist mit einem dreifach erhöhten Risiko für Herz-Kreislauf-Ereignisse verbunden.

### Koronarstenose

Zur standardisierten CT-morphologischen KHK-Klassifikation wird seit 2016 das CAD-RADS verwendet [5]. Die Einteilung erfolgt in 5 Klassen, basierend auf dem maximalen Stenosegrad:

- CAD-RADS 0: keine Plaque/Stenose
- CAD-RADS 1: 1–24 %
- CAD-RADS 2: 25–49 %
- CAD-RADS 3: 50–69 %
- CAD-RADS 4
  - 4A: 70–99 %
  - 4B: ≥ 70 % in allen 3 Gefäßen oder ≥ 50 % Hauptstamm
- CAD-RADS 5: 100 % (Gefäßverschluss)

Bei Patienten mit stabiler Angina pectoris gilt: Je höher die CAD-RADS-Klasse, desto höher ist das Risiko für zukünftige kardiovaskuläre Ereignisse. Unabhängig vom klinischen Risiko zeigt z. B. ein *CAD-RADS-3-Patient* ein 7‑mal höheres Risiko für das Auftreten eines kardiovaskulären Ereignisses im Vergleich zu einem *CAD-RADS-0-Patienten* [2].

Ein weiterer Vorteil der Klassifikation: Jede CAD-RADS-Klasse ist mit Empfehlungen für weitere diagnostische Maßnahmen und für das Patientenmanagement verbunden (Tab. 2). Die Behandlungsempfehlungen für Patienten mit akutem Koronarsyndrom variieren im Management und sind ebenfalls im Artikel zu CAD-RADS 2.0, der im Jahr 2022 überarbeiteten neuen Version der CAD-RADS-Klassifikation, beschrieben [5].

**Tab. 2:** Die 5 CAD-RADS-Klassen für Patienten mit chronischem Koronarsyndrom. (Nach [5])

	Maximaler Stenosegrad	Interpretation	Weitere Diagnostik	Weiteres Management
CAD-RADS 0	0 % (keine Stenose)	KHK-Ausschluss	Keine	Nichtatherosklerotische Ursachen erwägen
CAD-RADS 1	1–24 %	Minimale nichtobstruktive KHK	Keine	Risikomodifikation
				Risikomodifikation und präventive Therapie angepasst an *P*
CAD-RADS 2	25–49 %	Milde nichtobstruktive KHK	Keine	Risikomodifikation und präventive Therapie angepasst an *P*
CAD-RADS 3	50–69 %	KHK mit moderater Koronarstenose	Funktionstestung erwägen	Aggressive Risikomodifikation und präventive Therapie
				Antiischämische Pharmakotherapie erwägen
				Wenn Modifikator I+: invasive Koronarangiographie erwägen
CAD-RADS 4	A: 70–99 %	KHK mit hochgradiger Koronarstenose	A: Funktionstestung oder invasive Koronarangiographie erwägen	Aggressive Risikomodifikation und präventive Therapie
	B: ≥ 70 % in 3 Gefäßen oder ≥ 50 % Hauptstamm		B: Invasive Koronarangiographie	Antiischämische Pharmakotherapie erwägen
CAD-RADS 5	100 %	KHK mit totalem Verschluss einer Koronararterie	Invasive Koronarangiographie oder Vitalitätsdiagnostik erwägen	Weitere Behandlungen, inkl. Revaskularisation nach Leitlinien erwägen
CAD-RADS *N*	Nichtdiagnostische Untersuchungsqualität	Obstruktive KHK kann nicht ausgeschlossen werden	Zusätzliche/alternative Diagnostik erwägen	–

*CAD-RADS* Coronary Artery Disease—Reporting and Data System,* KHK* koronare Herzkrankheit, Modifikator *P* Plaquelast – insgesamte Menge an Koronarplaques, *Modifikator I* Ischämie

### Koronarsegmente

**Abb. 3:**
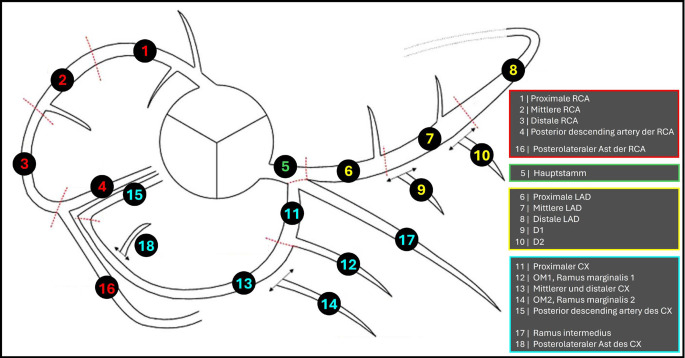
Der Koronarbaum zeigt die 18 Koronarsegmente der Society of Cardiovascular Computed Tomography (SCCT). *Gestrichelte Linien* repräsentieren die Grenzen der Koronarsegmente. *Rot* Segmente der *RCA* („right coronary artery“), *grün* Hauptstamm, *gelb* Segmente der *LAD* („left anterior descending“), *türkis* Segmente des Ramus circumflexus (*CX*). (Mod. nach [19])

Detektierte Stenosen werden den entsprechenden Koronarsegmenten zugeordnet. Hierfür eignet sich das 18-Segment-Modell der Society of Cardiovascular Computed Tomography (SCCT; [19]; Abb. 3).

### Modifikatoren

#### Modifikator N, S, G

Wenn ein oder mehrere Segmente z. B. aufgrund von Artefakten nicht beurteilbar sind, wird der Modifikator N (nichtdiagnostisch) verwendet. Dabei sollte beachtet werden, dass der Modifikator N nur dann als Zusatz im CAD-RADS-Ergebnis angegeben wird, wenn in einem anderen Segment eine obstruktive Stenose gefunden wird. Wenn keine obstruktive Stenose in den diagnostischen Segmenten gefunden wird, muss aufgrund des nichtdiagnostischen Segments bzw. der nichtdiagnostischen Segmente die gesamte Untersuchung als nichtdiagnostisch (CAD-RADS N; Tab. 2) eingestuft werden. Denn eine obstruktive KHK kann dann nicht ausgeschlossen werden.

Der Modifikator S steht für Stent, der Modifikator G für „Graft“ = Bypass.

#### Modifikator P

Ein weiterer wichtiger Faktor für das Ereignisrisiko ist die Plaque-Last, also das Ausmaß der KHK oder in anderen Worten die Gesamtanzahl der Plaques und betroffenen Gefäße. Die Plaque-Last wird durch die Graduierung von P1 (mild) bis P4 (extensiv) bestimmt. Sie basiert auf dem Kalziumscore oder der Anzahl der betroffenen Segmente (Tab. 3). Bei der Bestimmung der Plaque-Last ist es ähnlich wie beim Kartenspielen: Die höhere Zahl schlägt die niedrigere Zahl. Zum Beispiel wird ein Patient, der einen Kalziumscore von 50 hat, aber insgesamt 3 von Plaque betroffene Segmente aufweist, als „P2 – moderat“ eingestuft. Gut zu wissen: Die Plaquelast modifiziert die Empfehlungen zur Indikation und Stärke der Statintherapie in den CAD-RADS-Kategorien 1 und 2 ([5]; Tab. 2).

**Tab. 3:** Modifikator P: Kategorisierung der Plaquelast. (Nach [5])

	Plaquelast	Kalziumscore	SIS
P1	Mild	1–100	≤ 2
P2	Moderat	101–300	3–4
P3	Stark	301–999	5–7
P4	Extensiv	> 1000	≥ 8

*SIS* Segment Involvement Score, Anzahl der Segmente mit Koronarplaques

#### Modifikator I

Der Modifikator I (Ischämie) wird angegeben, falls eine Ischämie-Evaluation mittels CT-FFR oder myokardialer Perfusionsbildgebung verfügbar ist oder zusätzlich durchgeführt wurde. „I+“ wird angegeben, wenn Ischämie festgestellt wird, „I−“, wenn keine Ischämie nachgewiesen wird, und „I±“, wenn eine Ischämie grenzwertig vorliegt/nicht vorliegt. Der Modifikator kann auch nachträglich zum Befund hinzugefügt werden, falls der Patient nachfolgend noch eine Evaluation der CT-FFR oder eine funktionelle Bildgebung (z. B. Stress-Magnetresonanztomographie) des Herzens erhält.

#### Sonder-Modifikator HRP (High-Risk-Plaque)

Obwohl das akute Koronarsyndrom mit der Schwere der Stenose verbunden ist, sind die meisten Läsionen, die eine akutes Koronarsyndroms auslösen, nichtobstruktiv [4]. Unabhängig von der Schwere der Stenose und der Last an verkalkten Plaques sind sog. Hochrisikoplaques (HRP) Prädiktoren für das Auftreten eines akuten Koronarsyndroms und können helfen, das individuelle Risiko besser zu erfassen [26, 29]. HRP-Merkmale in der CT sind [21]:

- positives Remodeling
- niedrige Dichte < 30 HU (low attenuation“)
- fleckige Verkalkungen („spotty calcifications“)
- Serviettenring („napkin ring“)

**Abb. 4:**
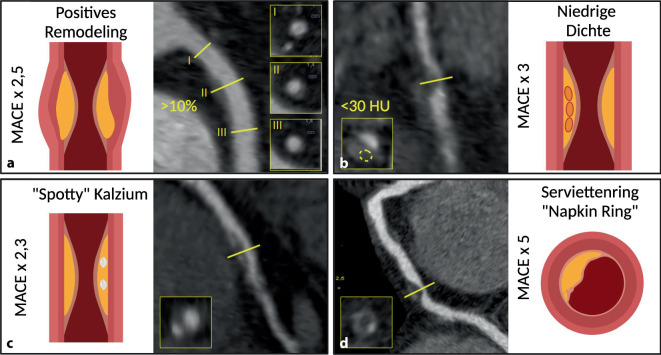
Beispiele für Hochrisiko-Merkmale koronarer Plaques in der Computertomographie-Koronarangiographie (CCTA) mit entsprechenden Querschnitten auf Höhe der Plaque (*gelbe Linien*). **a** Positives Remodeling (I und III: Referenzquerschnitte, II: Querschnitt auf Höhe der maximalen Stenose). **b** Plaque mit niedriger Dichte (< 30 HU, „low attenuation“). **c** Fleckige Verkalkungen („spotty calcifications“). **d** Serviettenring-Zeichen („napkin ring sign“). *MACE* Major Adverse Cardiac Events. (Created with BioRender.com)

Beispiele für Hochrisikomerkmale koronarer Plaques sind in Abb. 4 dargestellt. Wenn eine Plaque mindestens zwei HRP-Merkmale aufweist, wird der Modifikator „HRP“ im CAD-RADS-Ergebnis vergeben.

#### Modifikator E

Der Modifikator E („Exceptions“) bezieht sich auf Ausnahmen bzw. nichtatherosklerotische Abnormalitäten wie Koronardissektion, Koronaranomalie, Koronaraneurysma, Koronarfistel, arteriovenöse Malformationen und andere Ursachen. Diese Ausnahmen sollten separat im Befund beschrieben werden.

## CT-basierte fraktionelle Flussreserve

Die fraktionelle Flussreserve (FFR) ist ein Index, der eine Bewertung der hämodynamischen Relevanz einer koronaren Stenose ermöglicht. Traditionell wird die FFR in der invasiven Koronarangiographie mit Druckdrähten gemessen. Sie ist der Quotient des Blutdrucks proximal (in der Aorta) und distal der Koronarstenose. Fällt der Blutdruck distal der Stenose relevant ab, so wird auch der Quotient kleiner. Hierbei wird sich am Cut-off 0,80 orientiert [24]:

- FFR > 0,80: keine hämodynamisch relevante Stenose
- FFR ≤ 0,80: hämodynamisch relevante Stenose

**Abb. 5:**
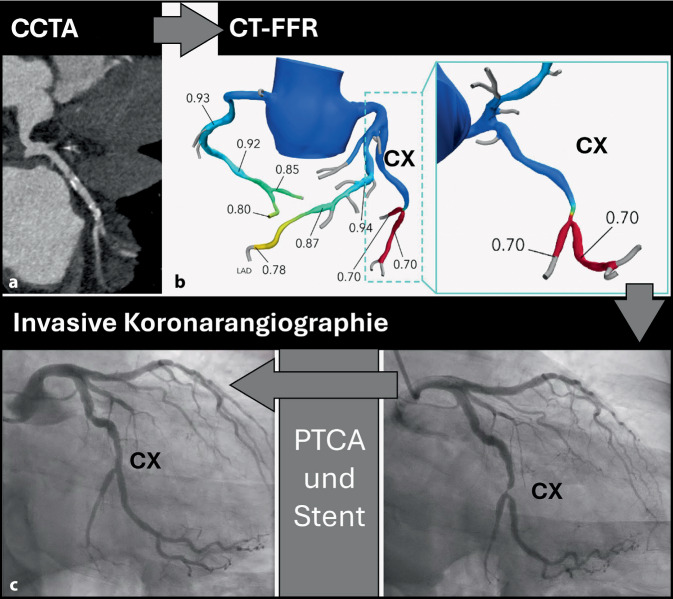
Ein 57-jähriger Mann mit pektanginösen Beschwerden wurde untersucht. **a** Die Computertomographie-Koronarangiographie (*CCTA*) zeigte eine schwere (70–99 %) Stenose im Ramus circumflexus (*CX*), CAD-RADS 4A. **b** Die CT-basierte Evaluation der fraktionellen Flussreserve (*CT-FFR*) zeigte Werte ≤ 0,80 und bestätigte somit die hämodynamische Relevanz der Stenose. **c** In der anschließenden Herzkatheteruntersuchung wurde eine Stenose von über 90 % im Ramus circumflexus/Ramus marginalis 2 festgestellt. Eine perkutane transluminale Koronarangioplastie (*PTCA*) wurde durchgeführt, bei der die Stenose mittels Implantation eines 3 × 18 mm-Stents behandelt wurde

Die CT-basierte FFR (CT-FFR) wird aus CCTA-Datensätzen berechnet und bietet eine nichtinvasive Alternative zum invasiven Goldstandard (Abb. 5) und wird laut aktuellen US-Leitlinien bei 40 %–90 %igen Stenosen empfohlen [13]. Sie bietet den Vorteil, Patienten potenzielle Risiken einer invasiven Prozedur zu ersparen. Die diagnostische Genauigkeit der CT-FFR wurde in mehreren multizentrischen Studien validiert und zeigt eine hohe Genauigkeit („area under the curve“ [AUC] 0,90; 95 % Konfidenzintervall [KI] 0,87–0,94) im Vergleich zum invasiven Referenzstandard [23, 25]. Die Integration der CT-FFR in klinische Diagnoseabläufe hat in zahlreichen Studien gezeigt, dass sie zu einer signifikanten Reduktion invasiver Koronarangiographien führt und somit zu einer Verbesserung der Lebensqualität der Patienten und zu Kosteneinsparungen [6, 8, 16]. Allerdings wurden die Kosten-Wirksamkeits-Studien im US-amerikanischen Raum durchgeführt. Es ist daher ungewiss, ob die aktuell hochpreisige CT-FFR (~1000 USD) im europäischen Raum eine ausreichende Vermeidung invasiver Tests erreicht, sodass die Gesamtbehandlungskosten entsprechend sinken würden. Derzeit werden neue Softwarelösungen entwickelt, die eine beschleunigte und kostengünstigere CT-FFR-Berechnung ermöglichen [3, 10]. Um eine Freigabe für die klinische Nutzung zu erlangen, müssen diese Lösungen jedoch noch validiert werden.

## CAD-RADS-Beispiel

**Abb. 6:**
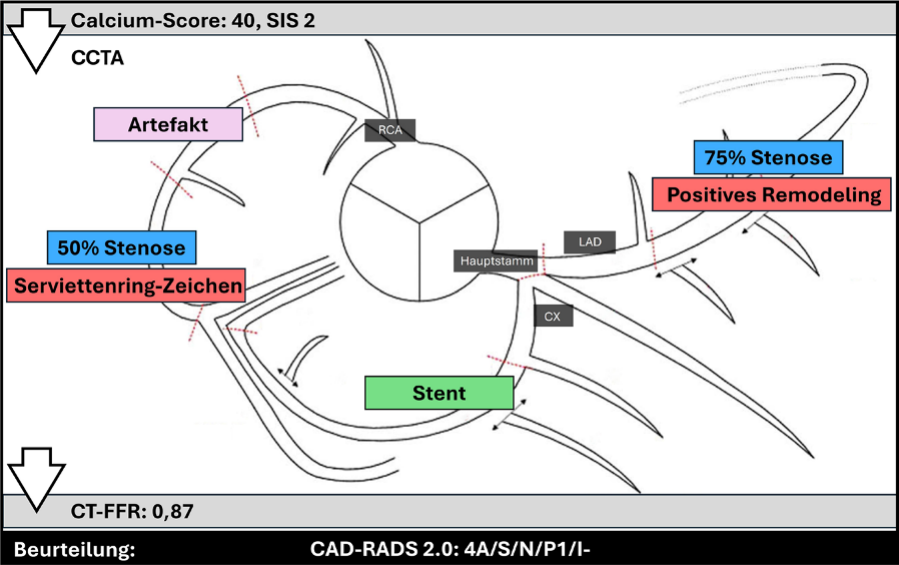
Beurteilung eines Beispiel-Patienten anhand der CAD-RADS‑2.0‑Klassifikation. Die schwerste Stenose (*blau eingefärbt*) befindet sich im Bereich der „left anterior descending“ (*LAD*) mit einem Grad von 75 %, was zu einer CAD-RADS-4A-Einstufung führt. Da beide Stenosen nur ein Hochrisikoplaque-Merkmal aufweisen, wird kein „Hochrisikoplaque (HRP)“ vergeben. Ein Stent (*grün eingefärbt*) ist im Stromgebiet des Ramus circumflexus (*CX*) zu sehen, daher wird der Modifikator „S“ vergeben. In der rechten Koronararterie (*RCA*) gibt es ein Segment, das aufgrund von Artefakten nicht beurteilbar ist, weshalb der Modifikator „N“ vergeben wird. Das vorangegangene Kalziumscoring ergab eine milde Koronarplaque-Gesamtmenge mit einem Score von 40 und zwei betroffenen Segmenten, daher wird „P1“ vergeben. Eine nachfolgende CT-basierte Untersuchung der fraktionalen Flussreserve (*CT-FFR*) ergab einen FFR-Wert von 0,87, was keinen Nachweis einer hämodynamisch relevanten Stenose darstellt; somit wird „I−“ vergeben. Die CAD-RADS‑2.0‑Beurteilung lautet: 4A/S/N/P1/I−. *SIS* Segment Involvement Score

In Abb. 6 wird anhand eines Beispiels veranschaulicht, wie die gesammelten Befunde der CCTA anhand der CAD-RADS‑2.0‑Klassifikation zusammengefasst werden.

## Fortschritte und Zukunft der CT-basierten Plaque-Analyse

In verschiedenen Bereichen der CCTA gibt es neue technologische Entwicklungen, die das Potenzial haben, in naher Zukunft zu einer weiter verbesserten individuellen Risikostratifizierung in der klinischen Routine beizutragen.

Bei der CT-Bildakquisition war die jüngste Einführung der Photon-Counting-Technologie eine bedeutende Entwicklung, die eine noch höhere Auflösung bei der Darstellung der Koronararterien ermöglicht [14].

Bei der Evaluation des koronaren Kalziums wurde kürzlich gezeigt, dass komplexe Merkmale der koronaren Verkalkungen mithilfe von maschinellem Lernen analysiert und in einem Radiomic-basierten Score ausgedrückt werden können, der als separater Biomarker dienen kann [9].

Bei der Untersuchung der Plaque-Zusammensetzung ermöglichen künstliche Intelligenz-gestützte Softwarelösungen eine (semi-)automatische, dichtebasierte Quantifizierung der verschiedenen Plaque-Bestandteile. Dieses Verfahren, auch bekannt als *virtuelle Histologie*, ermöglicht die Unterscheidung von nekrotischem Kern/Anteile niedriger Dichte (< 30 HU), fibrös-fettigen Anteilen (30 bis 129 HU), fibrösen Anteilen (130 bis 349 HU) und Kalzium (≥ 350 HU; [11]). Ergebnisse aus der SCOT-HEART Studie haben gezeigt, dass ein nekrotischer Kernanteil von über 4 % das 5‑Jahres-Ereignis-Risiko nahezu um das Fünffache erhöht, und das unabhängig von traditionellen Risikofaktoren und qualitativen Beurteilung der CCTA [29]. Die Quantifizierung der Plaque-Anteile und ihre Veränderung im Laufe der Zeit könnten genutzt werden, um den Verlauf der koronaren Herzkrankheit sowie beispielsweise das Ansprechen auf eine Statintherapie zu überwachen [30].

Dies könnte die Grundlage für eine potenziell verbesserte CCTA-basierte individuelle Prävention kardiovaskulärer Ereignisse bei asymptomatischen Patienten bilden, wie sie in der TRANSFORM-Studie Ende 2023 angekündigt wurde.

Zusammenfassend stellt die CCTA eine nichtinvasive, hochmoderne Diagnosetechnik dar, die eine detaillierte Erfassung, Charakterisierung und Quantifizierung von Plaques inklusive hämodynamischer Analyse erlaubt und somit eine Risikostratifizierung, die über das klinische Maß hinausgeht.

## Fazit für die Praxis

- Der Kalziumscore ist ein starker Ereignisprädiktor bei asymptomatischen Patienten (primäre Prävention), bietet jedoch nur eine grobe Information über das Ausmaß der koronaren Herzkrankheit (KHK).
- Hierbei ist es ratsam, die MESA-Perzentile zu berechnen.
- Die Computertomographie-Koronarangiographie (CCTA) wird bei Patienten mit intermediärem KHK-Risiko empfohlen, um das Risiko zu stratifizieren. Das Vorhandensein einer obstruktiven KHK (Stenose > 50 %) ist ein starker prognostischer Faktor.
- Die Präsenz von vulnerablen Plaques kann das Risiko weiter stratifizieren, vor allem in Patienten mit nichtobstruktiver KHK.
- Die Klassifikation des CAD-RADS (Coronary Artery Disease—Reporting and Data System) 2.0 fasst die wichtigsten Befunde standardisiert zusammen und gibt Therapieempfehlungen an.
- Die Charakterisierung von Plaques hat einen hohen prognostischen Wert bei asymptomatischen und symptomatischen Patienten.
- Quantitative Ansätze zur Messung von Volumina und Zusammensetzung der Plaques, unterstützt durch künstliche Intelligenz, gewinnen zunehmend an Bedeutung in der Forschung und werden in Zukunft voraussichtlich auch in der klinischen Praxis wichtig sein.
